# Suicidal ideation and attempts among high school students of war- affected area at Woldia town, Northeast, Ethiopia, 2022

**DOI:** 10.1186/s12888-023-04889-4

**Published:** 2023-05-31

**Authors:** Mulat Awoke Kassa, Mengesha Srahbzu, Goshu Nenko, Girum Nakie, Kindie Mekuria, Sefineh Fenta Feleke, Natnael Amare Tesfa, Berhanie Getnet

**Affiliations:** 1 Department of Nursing, College of Health Sciences, Woldia University, P. O. Box: 400, Woldia, Ethiopia; 2 Department of Psychiatry, College of Medicine and Health Sciences, University of Gondar, Gondar, Ethiopia; 3 School of Medicine, College of Health Sciences, Woldia University, Woldia, Ethiopia; 4 Department of Public Health, College of Health Sciences, Woldia University, Woldia, Ethiopia

**Keywords:** Suicidal ideation, Suicidal attempts, War, Mental health, Civilian mental heath, Demoralization, High school students, Ethiopia

## Abstract

**Background:**

Suicidal ideation and attempts usually occur during adolescence time, and living in war- affected area make the problem more predominate and severe. To the best of our knowledge, there were no studies done among high school students who live in war affected areas in Ethiopia.

**Objective:**

We assessed the prevalence and factors associated with suicide ideation and suicide attempts among high school students of war- affected area at Woldia town, Northeast, Ethiopia.

**Methods:**

School based cross-sectional study was conducted from May 23 to June 08, 2022.Data were collected from high school students in Woldia town, Ethiopia. Pretested, self-administered Amharic-language questionnaire was used to collect the data. Bivariable and multivariable logistic regression was used to identify the independent factors associated with suicide ideation and attempt.

**Results:**

A total of 668 of the 707 sampled students participated in the study (94.5% response rate). The prevalence of suicidal ideation and attempts among high school students in Woldia town was 16.29% and 12.87%, respectively. In the multivariable analysis, poor social support(AOR = 2.86, 95% CI:1.49, 5.46), posttraumatic stress disorder (AOR = 2.15, 95% CI:1.20, 3.85), family history of suicide(AOR = 3.94, 95% CI:2.21, 7.04), anxiety(AOR = 3.45, 95% CI:1.72, 6.89), and depression (AOR = 2.31, 95% CI:1.24, 4.33) were factors significantly associated with suicide ideation, and poor social support(AOR = 2.75, 95% CI:1.38, 5.47), depression (AOR = 4.27, 95% CI:2.10, 8.67) and being a female sex (AOR = 2.12, 95% CI:1.22, 3.69) were factors significantly associated with suicidal attempt.

**Conclusions and recommendations:**

This study revealed that at least one in six and one in eight of the students had suicidal ideation and attempt, respectively. Therefore, we recommend that Ministry of Education shall work with Ministry of Health to extend and implement mental health services in high schools and provide social support to those students who need the services in order for the prevention of suicidal ideation and attempts.

## Introduction

Suicide is defined as deliberately trying of killing oneself. It is described as a death caused by self-inflicted injury, poisoning, or suffocation in which the person planned to commit suicide and the injury was towards the self. Suicidal ideation (SI) is defined as any self-reported desire to harm oneself that is not accompanied by any preparatory behavior. Suicidal attempt(SA) is a nonfatal outcome that is instigated and perpetrated by the person in question and culminates in self-harm [1].

Suicide is among the top 20 causes of death for all ages worldwide. Every 40 s, one person commits suicide in the world [2]. Suicidal ideation and suicidal attempts usually occur during adolescence time [3]. In adolescence, young people frequently go through significant changes, learn new abilities, and encounter difficulties that can lead to suicide [4]. About 67,000 adolescents worldwide die by suicide each year [5]. Of this, over 79% of global adolescent suicides occur in low- and middle-income countries (LMICs) [6]. Even though the majority of suicide research is conducted in high-income countries, the data suggests that low- and middle-income countries have also this health threat. A study using Global School-Based Health Survey (GSHS) from 46 LMIC showed that the pooled 12-month prevalence of suicidal ideation and attempt were 14.5% and 12.7%, respectively [7]. A study high school students in 40 low- and middle-income countries (LMICs) reported a pooled mean 12-month prevalence rate for suicide attempt of 17.2% [8]. In Asia, suicide accounts for about 60% of World suicides, with China, India, and Japan accounting for about 40% of the World’s suicides [9]. A study from sub-Saharan countries using GSHS survey reported that suicidal ideation ranges from 11.2 -31.9% [10].

Living in war affected areas in which people directly or indirectly faced traumatic events, made suicidal ideation and attempts more predominate and severe. According to the Diagnostic and Statistical Manual 5 (DSM-5), a traumatic event includes exposure to actual or threatened death, serious injury or sexual violation [11]. Exposure to such an event is followed by psychological distress such as re-experiencing the event, avoiding associations with the event, negative cognitions, and mood, arousal, and the traumatic event eliciting emotional responses such as shock, denial, flashbacks and memory loss [11]. Studies have documented the prevalence of various traumatic experiences, their effects on people's physical and mental health, and how these effects might lead to suicidal ideation an attempt [12]. According to studies conducted in Lebanon, Afghanistan, and South Africa, between 50 and 70 percent of the population has experienced at least one traumatic event as a result of conflicts [13–15]. In Ethiopia there are also areas affected by traumatic events, for example, Woldia town, which is found in North Wollo in Northeast of Ethiopia, is one of the recently war-affected area in the last one year. High school students in this town were one of the demographic groups affected by the conflict's aftereffects. Most of the studies focusing on trauma and suicidality have been done in developed countries until now. Researches done in Bangladesh and Lebanon indicate that students who had traumatic experiences are more likely to have suicidal ideation [16, 17]. Increased suicidalities were discovered in African studies, for example, those conducted with students from war-affected places of South Africa and Uganda [18, 19].

Suicide and attempted suicide have detrimental emotional, physical, and financial effects. Suicidal attempt survivors may sustain severe trauma to themselves that have a long-term negative impact on their beings. Additionally, they could struggle with depression and other mental health problems and friends, relatives, coworkers of them, and the communities as a whole are affected*.* When someone commits suicide, their remaining relatives and friends may feel shock, angry, guilty, and show symptoms of depression and anxiety, or even have suicidal ideation of themselves [20]. Suicide costs society a lot of money as well. In terms of medical expenses, lost wages from employment, loss of statistical life, and quality of life expenditures, suicide, and nonfatal self-harm cost the country almost $490 billion in 2019 [21].

According to the studies, a number of factors affect high school students' suicidal ideation and attempts. Among these factors are PTSD, which has been shown to affect suicidal ideation in high school students in studies conducted in the United States and the United Kingdom [22–24], the interpersonal psychological theory of suicide stated that a combination of three interpersonal constructs such as thwarted belongingness (TB), perceived burdensomeness (PB) associated with sense of hopelessness about the possibility of change in these state and the third construct, acquired capability for suicide (AC) which is required condition before acting on the desire for suicide, are an important risk factors for suicide [25–28]. Other factors include female gender, lack of social support, loneliness, anxiety, being physically harmed, and the use of alcohol and other drugs [7, 29–34] and, school absenteeism were reported as a factor affecting suicidal ideation and attempt among high school students [31, 35, 36], disappointing with school results were also a factor that affect suicidal ideation and attempt among high school students [31, 37, 38]. In order to halt the threat of suicidal ideation and attempt, it is advisable to work on reducing risk factors and increasing protective factors and resilience among those students who live in war-affected areas. In these regards, different studies were done especially among developed countries, studies done in Ethiopia showed suicidal attempts of 12.5% and 16.2% in their life time respectively [31, 39], but these studies were not done in war affected areas, the current study assessed the prevalence and factors associated with suicidal ideation and attempt among high school students in the last 12 months after war affect in the area of Woldia.

## Methods and materials

### Study area and populations

An institutional-based cross-sectional study was conducted in May 2022 at four Secondary and High Schools of war-affected area in Northeast Ethiopia. The Schools are Woldia Comprehensive and Secondary school, Millennium Comprehensive and Secondary School, Genetie Comprehensive and Secondary School, and Mesenado Comprehensive and Secondary School.

The study area is situated in North Wollo Zone in the Amhara National Regional State with a distance of 521 km from Addis Ababa, the capital city of Ethiopia. A total of 46,139 people lived in the study area with 23,000 men and 23,139 women. Among the total population, 80.49% of the people were followers of Ethiopian Orthodox Christians religion, while 18.46% were followers of Muslim religion.

All high school students who attended their class during data collection time were included in this study. Whereas students who lived in the study area for less than one year and students who were unable to communicate due to acute illness during data collection time were excluded from this study.

### Sample size determination and procedures

The sample size was determined by assuming a single population proportion formula with the assumptions. The prevalence of suicidal ideation and attempt were 17.7% and 18.5% respectively [40], with 95% confidence interval (CI) and margin of error 2%, and 10% non-response rate. And then the final sample size for suicidal ideation was 683 and for suicidal attempt was 707, we have used 707 since it is higher than 668.

Before the actual data collection time, students were first stratified by their grade level as grade nine, grade ten, grade eleven, and grade twelve, considering each grade level as strata. The data we get from Education be rue of the study region showed that there were a total of 5100 high school students. Among these, grade nine accounts 1606, grade ten accounts 1230, grade eleven acounts1179, and grade twelve accounts 1085 of the students from the total high school students. Then, we made a proportional allocation for each stratum (grade levels) and as a result, 223 students from grade nine, 171 students from grade ten, 163 students from grade eleven, and 150 students from grade twelve were drawn. Finally, a computer generated lottery method using student’s identification number was applied to select study participants from each stratum. At the end, the selected students in each stratum were taken to one hall, and then the questioners were administered after orientation.

Data were collected using a structured self-administered questionnaire which includes: an outcome variable, suicidal ideation and attempt, which was assessed by using Composite international diagnostic interview (CIDI) with yes/no questions. Socio-demographic characteristics such as age, sex, and grade level were collected by using structured socio-demographic questionnaires. Clinical factors like family history of suicide, mental illness, and history of chronic medical illness were assessed using a structured yes/no questionnaire, history of anxiety, depression, and PTSD were assessed using GAD-7, PHQ-9, and PCL-5 respectively. Substance related factors, including Khat, tobacco, and alcohol which were assessed using a yes/no questionnaires adapted from the ASSIST (Alcohol,Smoking, and Substance Involvement Screening Test) [41, 42]. Finally, psychosocial factors including social support level, which was assessed using OSLO 3-items social support scale with scores ranging between 3 and 8 were classified as poor social support, a score between 9 and 11 as intermediate social support, and a score between 12 and 14 as strong social support level, and disappointed with school results, school absenteeism, having physical harm, were assessed using structured yes /no questions [43].

To control the quality of data, the questionnaire was translated appropriately into the local Amharic language. The training was given to data collectors and supervisors and each completed questionnaire was checked and the necessary feedback was also offered to data collectors following each morning. The questionnaire was pretested one week before the actual data collection time on 5% (*n* = 36) of the study participants who were not included in the main study.

The dependent variable assessment tool (PCL-5) had Cronbach alpha of 0.81. Based on the feedback obtained from the pretest, an appropriate modification was made to the questionnaire. The collected data were coded, edited, entered, and checked into the computer using EPI data version 4.6.02 and imported to STATA version 14.0 to generate descriptive statistics like means, standard deviation, frequency, and percentages. To determine an association between dependent and independent variables, adjusted odds ratios were used using logistic regression and the significance level was determined using a confidence interval of 95%. Bivariable and multivariable logistic regression was used to identify the independent predictors of PTSD. Each independent variable was separately entered in the bivariable analysis. Then variables with a *p*-value < 0.2 on bivariable analysis were entered into multivariate analysis. Then variables that showed a statistically significant association with a *p*-value < 0.05 on logistic regression were considered predictors of PTSD.

## Results

### Socio demographic characteristics of participants

Data were obtained from 668 high school students with a response rate of 94.5%. The mean age of the participants was 17.85 ± 1.658, ranging from 14 to 25 years old, and the majority of participants aged for 44.16% of participants were ranging between 14 and 18 years old. More than half the number (58.68%) of the participants was males, meanwhile 60.63% of them were living in urban areas as shown in Table 1 below.

**Table 1:** Sociodemographic characteristics of study participants

Variables	Categories	Frequency	Percent
Age	$<$ 18	295	44.1
	= 18	132	19.8
	$\ge$ 18	241	36.1
Sex	Male	392	58.7
	Female	276	41.3
Residency	Rural	263	39.4
	Urban	405	60.6
Father’s educational status	Unable to read and write	175	26.2
	Primary	243	36.4
	Secondary	126	18.9
	Higher education	87	13.0
	Non-formal	37	5.5
Mother’s educational status	Unable to read and write	225	33.7
	Primary	213	31.9
	Secondary	129	19.3
	Higher education	61	9.1
	Non-formal	40	6.0
Grade level	9	204	30.5
	10	182	27.3
	11	145	21.7
	12	137	20.5
Semester average score in percentage	$<70\%$	385	57.63
	70–84.5%	236	35.33
	$\ge$ 85%	47	7.04

### Clinical characteristics of the respondents

**Fig. 1:**
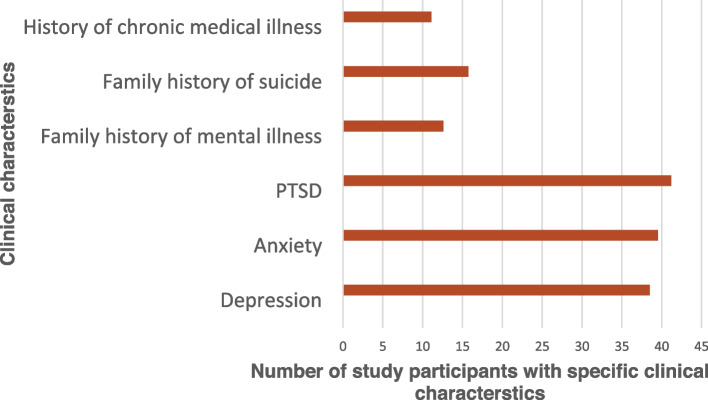
Shows clinical characteristics of study participants

Out of total participants, 41.1% had posttraumatic stress disorder, 11.1% of students had a history of chronic medical illness, and 12.6% of students had a family history of mental illness, 15.7% had a family history of suicide, 39.52% anxiety, and 38.5% depression as seen in Fig. 1.

### Substance related characteristics of participants

**Fig. 2:**
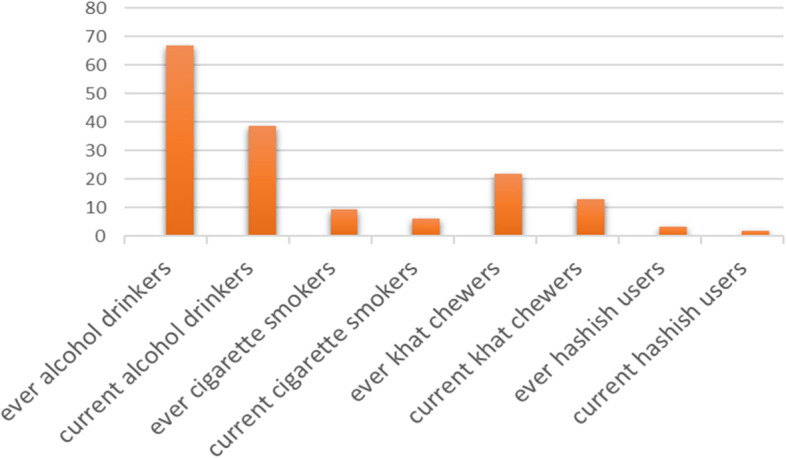
Shows substance -related characteristics of study participants and the number of study participants with specific substance related characteristics

Regarding substance use, out of the students 66.8% were ever alcohol drinkers, whereas khat and cigarette ever users were 22.1% and 9.3%, respectively, 38.6% of them were current alcohol drinkers as seen in Fig. 2.

### Psychosocial characteristics of participants

**Fig. 3:**
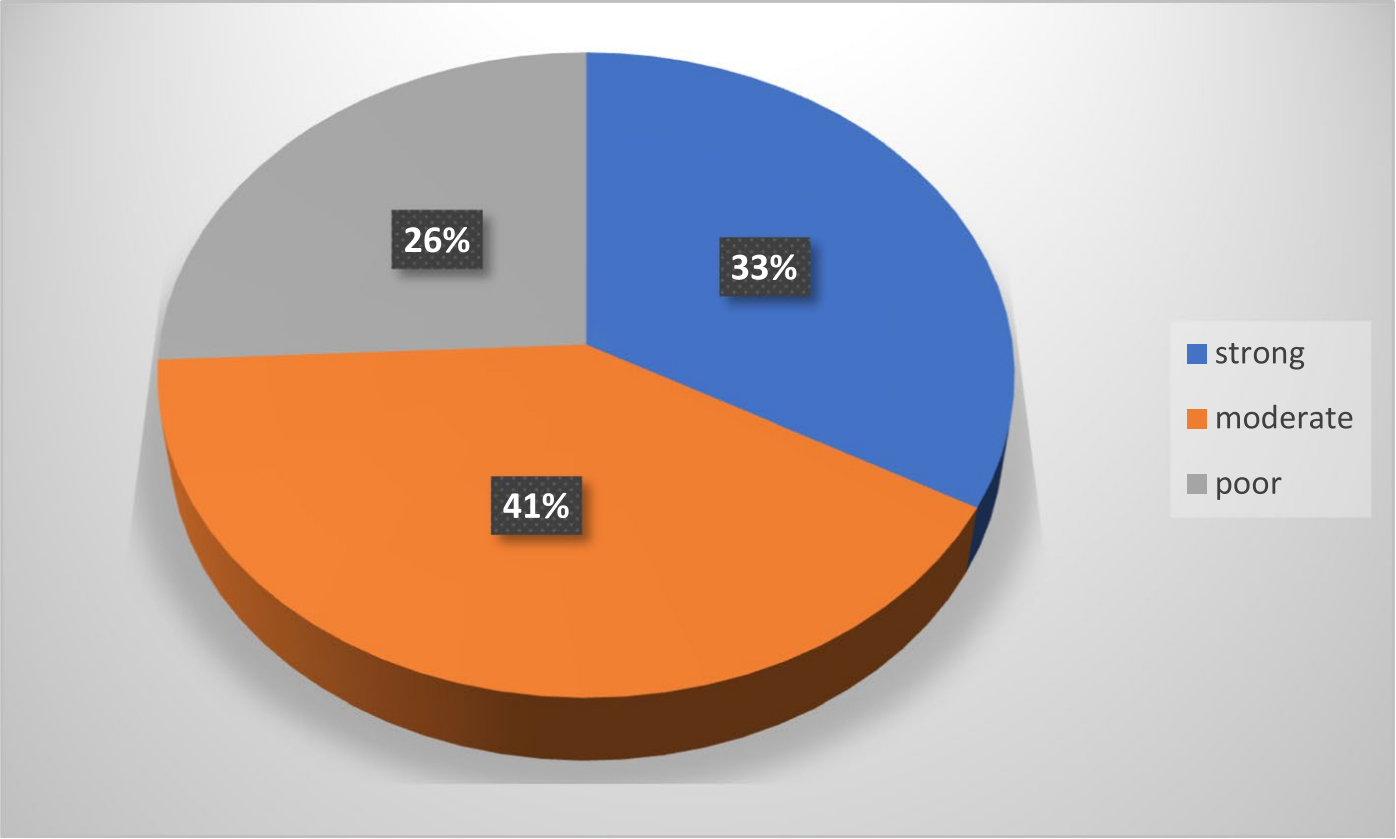
Shows social support level of study participants

From the participants, about one-third of students had strong social supports 33.1%, whereas students who had moderate and poor social supports were 41.2%, and 25.7%, respectively as seen in Fig. 3. Students who have been absented for greater than or equal to 4 days per month from school were 11.98%, and 12.87% of students experienced physical harm and 44.6% of the students were disappointed with their school results.

### Prevalence and associated factors of suicidal ideation and attempt

#### Prevalence of suicidal ideation and associated factors

In this study, the overall prevalence of suicidal ideation among high school students was 16.29% (95%, CI: 13.67%, 19.29%). Being female in sex, urban residency of students, having chronic medical illness, poor social support, having PTSD, having family history of suicide, ever and current alcohol drinking, having anxiety, and depression were factors associated with suicidal ideation at *p* < 0.2 in binary logistic regression.

Finally, in the analysis of multivariable logistic regression model findings revealed that having depression, family history of suicide, PTSD, anxiety and poor social support were found to be significantly associated with suicidal ideation with 95% of CI and at *p*- value < 0.05 as shown in Table 2 below.

**Table 2:** Bivariable and multi variable analysis of factors associated with Suicidal ideation among high school students in Woldia town, 2022 (*n* = 668)

Variables	Category	Suicidal ideation		COR (and 95% CI)	AOR (and 95% CI)
		Yes	No		
Sex	Female	59	217	1.85(1.23–2.81)	1.16(0.69–1.96)
	Male	50	342	1	1
Residency	Urban	76	329	1.61(1.03–2.50)	1.22(0.71–2.08)
	Rural	33	230	1	1
PTSD	Yes	84	191	6.47(4.00–10.45)	2.15(1.207–3.85) **
	No	25	368	1	1
Chronic medical illness	Yes	25	49	3.09(1.81–5.28)	0.85(0.39–1.84)
	No	84	510	1	1
Family history of suicide	Yes	46	59	6.18(3.88–9.86)	3.94(2.21–7.04) ***
	No	63	500	1	1
Social support	Poor	49	123	3.79(2.17–6.63)	2.86(1.49–5.46) ***
	Moderate	39	236	1.57(0.89–2.76)	1.39(0.74–2.62)
	Strong	21	200	1	1
Ever Alcohol drinking	Yes	29	366	1.45(0.91–2.30)	1.28(0.72–2.29)
	No	80	193	1	1
Current alcohol drinking	Yes	56	202	1.86(1.23–2.82)	1.62(0.93–2.85)
	No	53	357	1	1
Depression	Yes	83	174	7.06(4.39–11.36)	2.31(1.240–4.33) **
	No	26	385	1	1
Anxiety	Yes	90	174	10.48(6.19–17.73)	3.45(1.727–6.89) ***
	No	19	385	1	1

^*^ = *p*-value ≤ 0.05, ** = *p*-value ≤ 0.01, *** = *p* value ≤ 0.001

Hosmer -Lemeshow test = 0.75

Students who had poor social support were 2.86 times more likely to have suicidal ideation compared to students who had strong social support (AOR = 2.86, 95% CI: 1.49, 5.46).

Students who had a family history of suicide were about 3.9 times more likely to have suicidal ideation compared to those who had not a family history of suicide (AOR = 3.94, 95% CI: 2.21, 7.04).

Students who had PTSD were about 2.1 times more likely to have suicidal ideation than students who had not (AOR = 2.15, 95% CI:1.20, 3.85). Students who had anxiety were 3.5 times more likely to have suicidal ideation than students who had not anxiety (AOR = 3.45, 95% CI:1.72, 6.89).

Students who had depression reported 2.3 times higher suicidal ideation than students who had not depression (AOR = 2.31, 95% CI:1.24, 4.33).

#### Prevalence of suicidal attempts and associated factors

In the current study, the overall prevalence of suicidal attempts among high school students demonstrated 12.87% (95%, CI: 10.53%, 15.64%). Of those who attempted suicide, five methods of suicide attempt were used. Hanging was more used by boys (7.11%) than girls (6.32%) and poisoning was more (16.7%) used by the girls than boys (2.86%).

Jumping from high places and the use of sharp tools were less frequently used as means of suicidal attempts. Concerning the seriousness of the suicidal attempts, 54.6% of study participants report” I made a serious attempt to kill myself and it was only luck that I did not succeed” and 20.1% had attempted suicide to “cry for help”. Among attempters of suicide to cry for help, girls were higher than boys. Respondents gave a variety of reasons for their suicide attempt, among these family conflict, history of mental illness, and other reason than from response options were the most frequent reasons for suicidal attempts. Family conflict, and other reason from the response options were more frequently given as reasons for suicide attempts for the girls than boys, but having history of mental illness were the reason for suicidal attempts reasoned by both sexes equally.

Being female in sex, urban residency background, having chronic medical illness, poor social support, having PTSD, having family history of suicide, mental illness, current alcohol drinking, having anxiety, and depression were factors associated with suicidal attempt at *p* < 0.2 in binary logistic regression. Finally, the multivariable analysis model revealed that having depression, female sex, and having poor social support were found to be significantly associated with suicidal attempts with 95% of CI and at *p* < 0.05 as shown in Table 3 below.

**Table 3:** Bivariable and multi variable analysis of factors associated with Suicidal attempt among high school students in Woldia town, 2022 (*n* = 668)

Variables	Category	Suicidal attempt		COR (and 95% CI)	AOR (and 95% CI)
		yes	No		
Sex	Female	57	219	3.25(2.02–5.25)	2.12(1.22–3.69) **
	Male	29	363	1	1
Residency	Urban	63	342	1.92(1.15–3.18)	1.28(0.72–2.30)
	Rural	23	240	1	1
PTSD	Yes	24	213	4.47(2.71–7.38)	1.71(0.93–3.14)
	No	62	369	1	1
Chronic medical illness	Yes	21	53	3.22(1.82–5.68)	1.75(0.78–3.93)
	No	65	529	1	1
Family history of suicide	Yes	25	61	2.57(1.52–4.33)	1.43(0.70–2.92)
	No	80	502	1	1
Family history of mental illness	Yes	15	69	1.57(0.85–2.89)	0.51(0.20–1.32)
	No	71	513	1	1
Social support	Poor	41	131	4.01(2.16–7.43)	2.75(1.38–5.47) **
	Strong	16	205	1	1
Current Alcohol drinking	Yes	40	218	1.45(0.92–2.29)	1.44(0.83–2.48)
	No	46	364	1	1
Depression	Yes	70	187	9.24(5.22–16.34)	4.27(2.10–8.67) ***
	No	16	395	1	1
Anxiety	Yes	68	196	7.43(4.30–12.85)	1.74(0.84–3.61)
	No	18	386	1	1

^*^ = *p*-value ≤ 0.05, ** = *p*-value ≤ 0.01, *** = *p* value ≤ 0.001

Hosmer -Lemeshow test = 0.71

Students who had poor social support were 2.8 times more likely to have a suicidal attempt compared to students who had strong social support (AOR = 2.75, 95% CI:1.38, 5.47). Female students reported 2.1 times greater suicidal attempt than male (AOR = 2.12, 95% CI:1.22, 3.69). Students who had depression were 4.3 times more likely to have a suicidal attempts than students who had not depression (AOR = 4.27, 95% CI:2.10, 8.67).

## Discussion

The findings of the current study showed that the prevalence of suicidal ideation among high school students in Woldia town was 16.29% (95%, CI: 13.67%, 19.29%), which was inline with studies done in Ghana, Mozambique, US, middle and high income countries (M-HICs) from the six World Health Organization (WHO) regions, whose reported prevalence indicated 18.2%, 17.7%, 18.8%, and 14% respectively [35, 40, 44, 45].

However, the prevalence of suicidal ideation in this study was higher than previous studies done in Bangladesh, India, Thailand, with suicidal ideation of 9.3%, 5%, 8.8% respectively [38, 46, 47]. The possible reason for this difference may be due to the difference in socioeconomic and availability of health facilities and health professionals between those countries and Ethiopia in which factors like depression and other mental health risk factors of suicidal ideation may early detected and treated [48].

On the other hand, the current prevalence was lower than the prevalence of suicidal ideation found in the studies done in Benin, Poland, and Peru, which was 23.2%, 24.66% and 26.3% respectively [33, 49, 50]. The possible reason for this variation may be due to the difference in the composition of the study participant in the study, for example, in the study of Poland there were the greater proportion of female participants(70%) were involved in the study when compared with this study(41.3%) which might increase the risk of suicidal ideation [51].

The prevalence of suicidal attempts among high school students in this study was 12.87% (95%, CI: 10.53%, 15.64%), which was consistent with previous studies done in Bhutan and South Africa with a suicidal attempts of 11.3% and 14.8% respectively [51, 52].

However, the prevalence of suicidal attempts in this study was higher than previous studies done in Bangladesh, China, and United states of America, which showed the prevalence of suicidal attempt as 5.9%, 3.3% and 8.9% respectively [45, 53, 54]. The possible reason for this difference may be due to the difference in socioeconomic and availability of health facilities and health professionals between those countries and Ethiopia in which factors like depression and other mental health risk factors of suicidal ideation may early detected and treated [48].

On the other hand, the current prevalence was lower than the previous studies done in Benin, Liberia, Guatemala, and Mongolia, which found the prevalence of suicidal attempts as 28.3%, 33.7%, 16.6% and 32.2% respectively [32, 49, 55, 56]. The reason for this variation may be that in a study of Guatemala and Mongolia, there had been a greater number of female participants(more than 50%), but in the current study, females were lower than male participants 41.3%, and the risk of suicidal attempts was increased among female than males [57].

The odds of suicidal ideation were 2.86 times higher among high school students who had poor social support compared to those who had strong social support. This was supported by studies done in 46 low-and middle-income countries, Ethiopia and Malaysia [7, 29, 37]. The reason for this may be that social support makes a person feel that he/she is cared for and loved, regarded, and a member of a network of mutual duties which might decrease the risk of suicidal ideation [58]. Furthermore, social support can also aid people in coping with stressful situations and the challenges brought on by psychopathology, which may lower the risk of suicide [58]. Thus, indicating that social support may be particularly valuable for helping the most vulnerable individuals.

The odds of having suicidal ideation were 2.1 times higher among students who had posttraumatic stress disorder (PTSD) compared to those who had not posttraumatic stress disorder (PTSD). Studies had also been revealed results [22–24, 59]. The reason for this could be that those with PTSD are more likely to experience suicidal ideation because they have trouble controlling their emotions and their urges [60]. The other reason for this may be that sometimes those who suffer from PTSD also struggle with despair, panic attacks, extreme anxiety, or substance abuse, this could increase the chance of suicidal ideation, and individuals might believe that the only option is to commit suicide [61]. Hence, we have to give concern about the symptoms of PTSD by having treatment options like either PE (prolonged exposure) or CPT (cognitive processing therapy) [62]

The odds of having suicidal ideation were 3.9 times more likely among students who had a family history of suicide compared to those who had not a family history of suicide. This finding was supported by studies done in different countries [39, 63–65]. The reason for this could be a genetic etiology [66].The other possible reason may be that mental health problems, such as depression, are inherent, which may increase the risk of suicidal ideation on off spring [67]. This suggests emphasizing students with a family history of depression or suicide.

Students who had anxiety were 3.5 times more likely to have suicidal ideation compared to their counter parts. This was supported by studies done in Malaysia, and Mozambique [40, 68]. The reason for this may be that a person with anxiety symptoms faces an intolerance of the pain that anxiety causes, especially, when /she reaches the level of panic, and a person may have suicidal ideation as a solution to escape from this pain. Another possible reason could be that individuals with anxiety symptoms have an increased risk of having comorbidities with depression, which has a direct effect on suicidal ideation [69]. This finding suggested that working on anxiety is paramount to reduce suicidal ideation. We may have the following means of managing anxiety disorder such as learning about anxiety, mindfulness, relaxation techniques, correct breathing techniques, dietary adjustments, exercise, learning to be assertive, building self-esteem, cognitive therapy, exposure therapy, structured problem solving, medication and support groups (https://www.betterhealth.vic.gov.au/health/conditionsandtreatments/anxiety-treatment-options#managing-anxiety).

Students who had depression were 2.3 times more likely to have suicidal ideation as compared to those who had not depression. This was supported by studies done in Sub Saharan Africa, in Zambia, Tunisia, and Nigeria and in Thailand [47, 70–72]. The reason for this could be that the direct impact of depression, which makes people feel hopeless and worthless, which in turn increases the risk of suicidal ideation [73]. Hence, giving priority for depressed individual is vital means to reduce suicidal ideation. Here are typical examples to reduce depression; practicing self-care such as getting regular sleep and exercise, and maintaining healthy nutritional practices; having open, honest conversations about what you or someone else with depression with suicidal ideation may be experiencing and feeling; learning and recognizing the warning signs of depression with suicidal ideation (https://www.healthline.com/health/depression/suicidal-depression).

The odds of suicidal attempts were 2.8 times higher among students who had poor social support compared to those who had strong social support. This was supported by studies done in 46 low-and middle-income countries and two studies done in Ethiopia [7, 31, 37]. The reason for this could be due to that those with poor social support feel that their existence was a burden on others such as family members. In addition, social support can refer to the presence of those who can aid people in coping with stressful situations and the challenges brought on by psychopathology, which may lower the risk of suicidal attempt [58]. Therefore, comprehensive MHPSS programs for war-affected people that integrate somatic health concerns, social support, education, and targeted psychiatric/psychological interventions are urgently needed [74].

Students who had depression were 4.3 times more likely to have suicidal attempts compared to their counter parts. Studies done in Ethiopia, India, Tunisia and Nigeria were also revealed this [37, 38, 70, 71]. The reason behind this may be that it was indicated that the decreased level of serotonin neurotransmitters in the brain of a depressed individual was found to be associated with increased suicidal attempt [75]. The other reason for this could be that the direct impact of depression, which makes people feel hopeless and worthless, which in turn increases the risk of suicidal attempt [73]. In addition, peoples may have symptoms like hopelessness, helplessness, giving up, feeling of isolation, and despair which were the core features of demoralization which in turn leads to suicidal attempts [76].

The odds of having suicidal attempts were 2.1 times more likely among female students than males. This was supported by studies done in Ethiopia and Benin [7, 37, 49]. The reason for this association may be due to the fact that gender related vulnerability to psychopathology and to psychosocially disadvantageous circumstances. More specifically, depression is the most common risk factor for serious suicidal attempts in both men and women, but happens twice as often in girls as in men. This implies that great emphasis should be given for females.

## Limitations of the study

This study is conducted with some limitations. Since it was cross-sectional study, it could be difficult to declare cause-and effect relationship between suicidal ideation and attempt and factors like social support, depression, PTSD, and anxiety in addition, recall bias may occur during data collection since the study collected retrospective data of 12 months. Furthermore, we did not use a specific scale to assess suicidal ideation such as Beck's scale. Beck’s scale contain 19 items and each item is scored from 0 to 2 in ascending order of.severity, giving a total score ranging from 0 to 38 [77].

## Conclusions and recommendations

This study revealed that at least one in six of the high school students in the sample had experienced suicidal ideation and one in eight had attempted suicide. The distribution of suicidal ideation among high school students showed that it was higher in students with PTSD, anxiety, depression and among those who have a family history of suicide and poor social support. The current study had also showed that suicidal attempt was higher among female students, students who had poor social support, and students who had depression. Therefore, we recommend that Ministry of Education shall work with Ministry of Health to extend and implement mental health services in high schools and provide social support to those students who need the services in order for the prevention of suicidal ideation and attempts.

## Data Availability

Data is available upon request from the corresponding author.
